# Reversal of Multidrug Resistance in Cancer by Multi-Functional Flavonoids

**DOI:** 10.3389/fonc.2019.00487

**Published:** 2019-06-12

**Authors:** Qingmei Ye, Kai Liu, Qun Shen, Qingyue Li, Jinghui Hao, Fangxuan Han, Ren-Wang Jiang

**Affiliations:** ^1^Hainan General Hospital, Haikou, China; ^2^Jiaozuo Second People's Hospital, Jiaozuo, China; ^3^Guangdong Province Key Laboratory of Pharmacodynamic Constituents of TCM and New Drugs Research, Cooperative Laboratory of Traditional Chinese Medicine Modernization and Innovative Drug Development of Chinese Ministry of Education, Jinan University, Guangzhou, China

**Keywords:** multidrug resistance, natural products, flavonoids, overcome, cancer, drug discovery

## Abstract

Multidrug resistance (MDR) resulting from different defensive mechanisms in cancer is one of the major obstacles of clinical treatment. To circumvent MDR many reversal agents have been developed, but most of them fail in clinical trials due to severely adverse effects. Recently, certain natural products have been reported to overcome MDR, including flavonoids which are abundant in plants, foods, and herbs. The structure of flavonoids can be abbreviated as C6-C3-C6 (C for carbon), and further categorized into flavonoids, iso-flavonoids and neo-flavonoids, according to their structural backbones. Flavonoids possess multiple bioactivities, and a growing body of research has indicated that both flavonoids and iso-flavonoids can either kill or re-sensitize conventional chemotherapeutics to resistant cancer cells. Here, we summarize the research and discuss the underlying mechanisms, concluding that these flavonoids do not function as specific regulators of target proteins, but rather as multi-functional agents that negatively regulate the key factors contributing to MDR.

## Introduction

Multidrug resistance (MDR) is one of the major challenges in cancer treatment (1), which occurs in a short period of time during/after treatment, and may result in cross resistance to many other structurally and mechanically different chemotherapeutics (2). MDR may be due to different mechanisms, including (1) ATP-binding cassette (ABC) transporters that pump out chemotherapeutics (3), (2) the mutation of oncogenes that become resistant to former treatments (4, 5), (3) an evolving adaptation of cancer cells to the microenvironment (6, 7), (4) survived cancer stem cells (CSCs) that escape from conventional therapies (8, 9), and (5) activated cell growth factors as well as cell defense systems, etc.

As membrane-bound proteins, ABC transporters refer to 49 transporter proteins that are classified into seven subfamilies, ABCA to ABCG, that locate in the cell membrane and have diverse functions (10). ABC transporters have two nucleotide-binding domains (NBDs) which bind and hydrolyze ATP, and two trans-membrane binding domains (TMDs) which carry their substrates out of the cell (11, 12). By using ATP, ABC transporters work to transport their substrates across the cell membrane, and the substrates include building blocks/nutrition such as amino acids, sugars, lipids, vitamins, peptides, and certain proteins etc. Importantly, they can protect cells against xenobiotics, including some anti-cancer drugs (13). Higher expressions of these transporters, such as ABCB1 (also known as P-glycoprotein, P-gp), ABCG2 (also known as breast cancer resistant protein, BCRP), and ABCC1 (also known as multidrug resistance-associated protein 1, MRP1), have closely participated in MDR as confirmed by studies from both the laboratory and the clinic (14, 15). The overexpression of ABC transporters may lead to the resistance of conventional chemotherapeutics, such as doxorubicin (Dox), paclitaxel, colchicine, etc., radiotherapy, and targeted therapies, such as imatinib (14).

Cancer cells may also adapt to the changed microenvironment, e.g., the increased oxidative stress, leading to MDR. Oxidative stress is defined as the phenomenon of imbalance between the production of reactive oxygen species (ROS) and antioxidant defenses, which plays a key role in the initialization of many diseases for their impacts on tissue damage (16). Oxidative stress also contributes to tumor development and responses to anticancer therapies (17). Generally, certain level of ROS may benefit cancer cell proliferation and DNA mutations, while high level ROS may be a lethal factor that finally induces cell death (18). Research has shown that ROS levels are higher in cancer cells and in resistant cancer cells due to chemotherapy or radiotherapy (17, 19). Accordingly, the corresponding antioxidant pathways that eliminate ROS are up-regulated during tumor initiation and progression, rendering them more vulnerable to further oxidative stress assaults (18, 20). Therefore, targeting oxidative stress is a promising strategy to overcome MDR in cancer.

Cancer cells that grow rapidly need more oxygen supply for their energy supply and signal transmission (21, 22). Tissue hypoxia occurs due to an inadequate amount of oxygen delivery or due to cancer cell metabolism re-programming, rendering cancer cells to adapt to less oxygen by up-regulating several key proteins, including hypoxia-inducible factor-1α (HIF-1α), HIF-2α (23). More importantly, hypoxia can trigger MDR by impacting the efficacy of anticancer drugs (24). Furthermore, hypoxia may also induce the expression of ABCB1 and ABCG2 that pump out intracellular chemotherapeutic agents (25, 26), a common MDR mechanism.

Cancer stem cells (CSCs), a subset of cells within the tumor, that possess the potential of self-renewal, differentiation and tumorigenicity, are thought to be the major cause of cancer therapy failure due to their chemo- and radio-resistance (9, 27). CSCs are situated in the niche, which are mainly composed of fibroblasts and endothelial, mesenchymal and immune cells, playing pivotal roles in drug resistance (28). Therefore, the elimination of CSCs represents one promising strategy to overcome MDR.

The cell cycle, the mechanism of cell division, is composed by four phases: the G1 phase, during which a cell begins to grow in size to be ready to DNA synthesis; the S phase (synthesis), during which cell synthesizes DNA; the G2 phase, during which a cell continues to grow to be ready for mitosis; the M phase (mitosis), during which the cell stops growing and divides into two cells (29, 30). The cell cycle is driven by cyclin-dependent kinases (CDKs) which are regulated by cyclins (cyclin A-Y). Studies have shown that certain phases of the cell cycle exhibit resistance to chemotherapeutics (31, 32), and cancer cells that over-express CDKs and cyclins demonstrate resistance to conventional chemotherapeutics (33–35).

Autophagy, a self-degradative system in which cells undergo degradation of intracellular components, is important for the energy balance in response to nutrient stress (36, 37). During chemotherapy, autophagy works as a prosurvival and resistance mechanism; therefore, the inhibition of autophagy can re-sensitize MDR cells and enhance the cytotoxicity of chemotherapeutic agents (38).

Epithelial mesenchymal transition (EMT), a biologic process that polarized epithelial cells undergoes multiple biochemical changes to achieve mesenchymal cell phenotype including enhanced metastasis, invasiveness, drug resistance (39, 40), which play an important role in the morphogenesis of multicellular organisms (41).

Other key enzymes in cancer cells are also overexpressed to evade the cell death induction caused by chemotherapeutics. Signal transducer and the activator of the transcription (STAT) protein family (STAT 1-6) are intracellular transcription factors that mediate cellular differentiation, proliferation, hematopoiesis, and apoptosis by transmitting signals from the cell surface receptors to the nucleus (42). STAT3 plays a pivotal role in tumor growth and metastasis and it is activated and up-regulated in solid tumors and resistant cancers, suggesting it as a promising target to overcome MDR (43–45).

p53 (also known as TP53) is a classic tumor suppressor gene that induces cell cycle arrest and apoptosis (46). Usually, p53 is down-regulated or mutated in cancer cells, especially in the cancer cells of MDR (47).

Another key player, the nuclear factor kappa-light-chain-enhancer of activated B cells (NF-κB), composed with five transcription factors, can bind to DNA sequences at promoter regions of responsive genes to regulate cellular processes such as DNA transcription, cytokine production, and cell survival (48). Activated NF-κB not only promotes tumor cell proliferation and apoptosis suppression, but it also induces EMT which facilitates distant metastasis and drug resistance (49, 50).

Various MDR reversal agents have been developed and some of them have entered into clinical trials, however, most of them failed due to severely adverse effects or because they suffered resistance in a short time (51, 52). Effective novel agents that surmount MDR remain an unmet clinical need.

**Figure 1:**
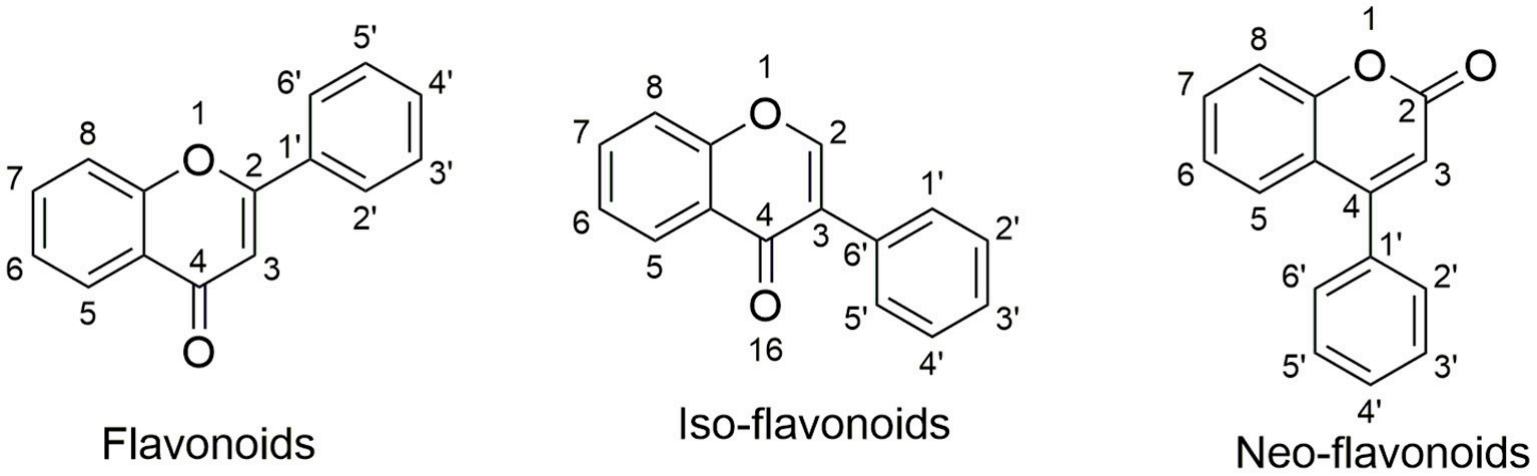
Structural backbones of flavonoid, iso-flavonoid, neo-flavonoid.

Natural products are the major resource for new lead compound identification and new drug discoveries, which account for nearly 50% over the past three decades (53). Of all the versatile chemical structures, flavonoids are one of the most intensively studied. Flavonoids are abundant in plants, foods such as fruits and vegetables, as well as in traditional herbs (54–56). Importantly, many flavonoids have been applied in humans for nutrition supply and for certain disease treatment (57, 58), indicating their safety properties. Structurally, flavonoids are classified into three categories: flavonoids; iso-flavonoids; neo-flavonoids, as shown in Figure 1. Specifically, flavonoids have a backbone of 2-phenyl-1,4-benzopyrone, iso-flavonoids have a backbone of 3-phenyl-1,4-benzopyrone, and neo-flavonoids have a backbone of 4-phenyl-1,2-benzopyrone. To date, ~5,000 diverse flavonoids have been identified (59).

**Figure 2:**
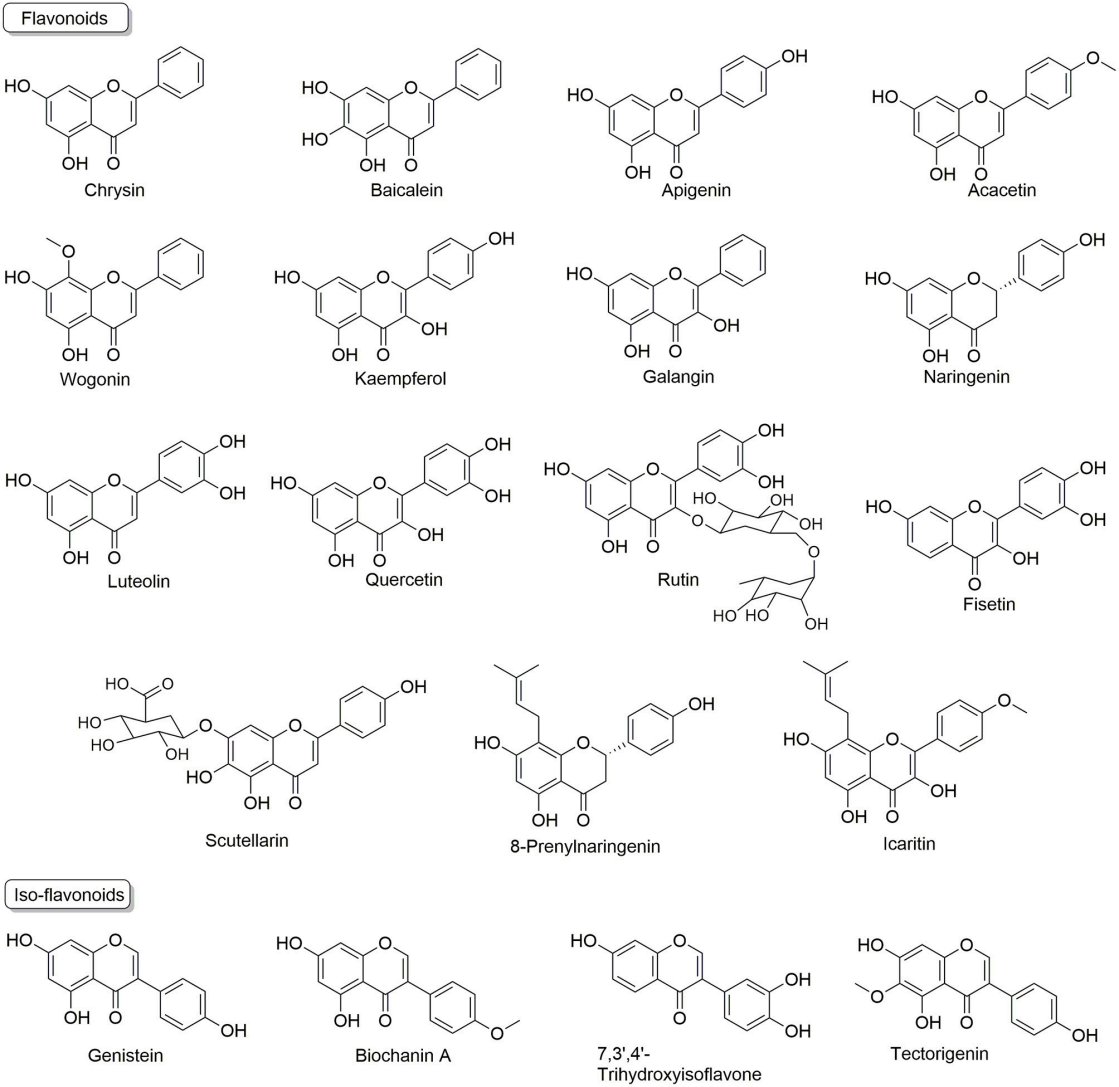
Chemical structures of flavonoids and iso-flavonoids that have MDR reversal effects.

Flavonoids are usually termed as multi-targeting and multi-functional molecules, as they possess multiple bioactivities, such as cardiovascular protective effects (60), nerve system protective effects (61), anti-aging (62), anti-inflammatory (63), anti-cancer (64), so on and so forth. More importantly, flavonoids (as summarized in Figure 2) have been found to kill resistant cancer cells or to re-sensitize conventional anti-cancer drugs to reverse MDR via the mechanisms discussed above, indicating their appealing potential in resistant cancer treatment. Here, we summarize these reports and discuss the analyzing of underlying mechanisms.

## Multi-Functional Flavonoids Overcome MDR in Cancer

### Flavonoids That Regulate ABC Transporters to Overcome MDR

Many flavonoids, such as Chrysin, Baicalein, Kaempferol, Quercetin, Rutin, Icaritin, and iso-flavonoids, such as Genistein and Biochanin A, have been found to regulate ABCB1, ABCG2, ABCC1 and other transporters to reverse MDR.

**Chrysin**, 5,7-dihydroxyflavone, which presents in honey, propolis, and the passion flower *Passiflora caerulea* (65), exhibits various bioactivities, including anti-cancer effects as it is reported to inhibit aggressive anaplastic thyroid cancer cells (66) and drug resistant triple-negative breast cancer cells (TNBC) (67). Chrysin can inhibit ABCB1 mediated rhodamine 123 (an ABCB1 substrate) efflux on human breast cancer cells MDA-MB-231 (68). Chrysin may also regulate ABCG2 mediated nitrofurantoin transport on ABCG2-overexpressing human MCF-7 breast cancer cells by increasing the area under the curve (AUC) (69). Moreover, Chrysin sensitizes the ABCG2-transfected cells to mitoxantrone (an ABCG2 substrate) via stimulating ATPase (70).

**Baicalein**, 5,6,7-Trihydroxyflavone, isolated from *Scutellaria baicalensis* and *Scutellaria lateriflora* (71), holds potential in treating breast cancer (72), colorectal cancer (73), bladder cancer (74), etc. Baicalein may reverse ABCB1 mediated MDR as shown on ABCB1 gene transfected Madin-Darby canine kidney II (MDCK II) cells (75). Baicalein induces apoptosis and autophagy and decreases ABCB1 and anti-apoptotic Bcl-xl expression levels on 5-fluorouracil (5-FU) and Epirubicin resistant hepatocellular carcinoma cells (Bel7402/5-FU). By inhibiting an ABCB1-mediated drug efflux, Baicalein (5 g/ml and 10 g/ml) increases the intra-cellular concentrations of rhodamine 123 and Epirubicin (76). Through this similar mechanism, Baicalein enhances the cytotoxic effects of docetaxel in anaplastic thyroid cancer 8505c cells (77), and paclitaxel in its resistant MCF-7/Tax cells and in an animal model (78).

**Apigenin**, 4′,5,7-Trihydroxyflavone, isolated from Apium graveolens (79), shows anti-cancer effects to human breast cancer (80), prostate cancer (81), and imatinib-sensitive and resistant chronic myeloid leukemia K562/IMA3 cells (82). One molecular docking study indicates that Apigenin binds to the NDBs of ABCB1 and ABCB5 (83). Apigenin inhibits ABCB1 expression and re-sensitizes docetaxel-resistant prostate cancer DU145 cells to docetaxel (84). Through down-regulating ABCB1, Apigenin (2, 8 μM) significantly enhances the efficacy of doxorubicin (Dox, an ABCB1 substrates) in its resistant MES-SA/Dx5 cells (85) and breast cancer cells (MCF7/ADR) (86).

**Acacetin**, O-methylated Apigenin, found in *Robinia pseudoacacia, Turnera diffusa*, and *Betula pendula* (87), exhibits anti-cancer effects in prostate cancer cells (88) and hepatocellular carcinoma (89) etc. Acacetin inhibits the activities and functions of both ABCB1 (90, 91) and ABCG2 (92). Through down-regulating ABCB1 in non-small cell lung cancer (NSCLC) cells, Acacetin decreases efflux of Dox by 59% and further increases accumulation of Dox inside the cells up to 55%, leading to synergistic cytotoxic effects (91). As an ABCG2 inhibitor, Acacetin potentiates the cytotoxicity of SN-38 and mitoxantrone (both are ABCG2 substrates) in ABCG2-transfected K562 (K562/BCRP) cells (92).

**Wogonin**, 5,7-dihydroxy-8-methoxyflavone, isolated from *Scutellaria baicalensis* (93), exhibits multiple anti-cancer effects to gastric cancer cells, lung cancer cells and glioma cancer cells (94–96). Wogonin appears to be an inhibitor of ABCB1 (97), and it suppresses the function of ABCB1 and increases the cellular content of etoposide in HL-60 cells (98). In Dox-resistant human myelogenous leukemia K562/A02 cells, Wogonin re-sensitizes Dox by inhibiting functional activity and expression of ABCB1 at both protein and mRNA levels (99).

**Kaempferol**, 3,4′,5,7-tetrahydroxyflavone, a secondary metabolite found in many plants, plant-derived foods, and traditional medicines (100), possesses inhibitory activities to gastric cancer cells, lung cancer cells (101, 102), and tyrosine kinase inhibitor (TKI)-resistant lung cancer cell line H1993 (103). Kaempferol can inhibit the efflux of ABCB1 via stimulating ATPase activity (104, 105). It is also an ABCG2 substrate and it suppresses ABCG2 up-regulation (106), indicating its potential as a reversal agent. Indeed, Kaempferol (20 μM) shows a synergistic efficacy with cisplatin in surmounting ovarian cancer OVCAR-3 cells, and the combination inhibits the mRNA levels of ABCC6 and cMyc (107).

**Naringenin**, 4′,5,7-Trihydroxyflavanone, present in many fruits, and herbs (108), exhibits inhibitory effects to prostate cancer cells and glioblastoma cells (109, 110). Naringenin may inhibit the efflux of ABCB1 (111) via interactions with the hydrophobic pocket of the transporter as confirmed by a docking study (111). Through this mechanism, Naringenin significantly enhances the cytotoxicity of daunomycin to resistant human breast cancer cell lines MCF-7/ADR cells (112).

**Quercetin**, found in many fruits, vegetables, leaves, and grains, has been used as a nutrition supply for many years (113). Quercetin has been reported to kill many types of cancer cells, including human breast cancer MCF-7 cells (114), NSCLC A549 cells (115), ovarian cancer cells (116), etc. Quercetin is able to block the function and expression of ABCB1 and ABCC1, ABCC2 (112, 117, 118). As tested in 5-FU resistant human hepatocellular carcinoma BEL/5-FU cells, Quercetin inhibits the functions and down-regulates the expressions of ABCB1, ABCC1, ABCC2 (118). Quercetin is found to inhibit the pumping effects of these three transporters, evidenced by more intracellular accumulation of rhodamine-123 and Dox (118). On ABCB1 over-expressing and Dox resistant human breast cancer MCF-7/dox cells, Quercetin significantly enhances the antitumor activity of Dox, paclitaxel, and vincristine. The combined treatment of Dox, paclitaxel, and vincristine with Quercetin significantly down-regulates ABCB1 expression and eliminates breast cancer stem cells (119). Further studies also confirms the reversal effects of Quercetin (0.7 and 25–100 μM, respectively) in MCF-7/dox cells (120) and in gene-encoded ABCB1 overexpressing oral cancer KB/VCR cells (121).

Other flavonoids that regulate ABC transporters include Rutin, a quercetin glycoside that inhibits the pumping effects of ABCB1 and ABCG2 (117, 122), Fisetin, found in many that has been found to possess sensitizing effects to conventional chemotherapeutics cabazitaxel (123) and paclitaxel or arsenic trioxide in NSCLC (124), 3,3′,4′,7-Tetrahydroxyflavone, found in many fruits and vegetables (125), inhibits the function of ABCB1 (85), as well as 8-Prenylnaringenin (126), a prenylflavonoid phytoestrogen found in hops (*Humulus lupulus*) and beer (127), and a clinical drug candidate Icaritin (under clinical trials in China for treatment of hepatocellular carcinoma), both of which inhibit the efflux of ABCB1 and ABCC1 (128, 129).

In addition, there are two iso-flavonoids that regulate ABC transporters. **Genistein**, 4′,5,7-Trihydroxyisoflavone, found in a number of plants including lupin, fava beans, soybeans, is an angiogenesis inhibitor that exhibits anti-cancer activities (130). Genistein is also reported to be an inhibitor of ABCB1, ABCG2 (131, 132). As tested in ABCG2-transduced MDCK-II cells, Genistein can inhibit the transport of Danofloxacin, a substrate of ABCG2 (133). Other further applications to reverse MDR mediated by ABC transporters remain to be explored.

**Biochanin A**, 5,7-Dihydroxy-4′-methoxyisoflavone, found in soy, exerts certain anti-cancer effects (134, 135). Biochanin A is found to be an ABCG2 inhibitor, as it increases the accumulation and cytotoxicity of mitoxantrone in mitoxantrone resistant MCF-7 MX100 cells which over-express ABCG2 (136).

### Flavonoids That Regulate Oxidative Stress to Overcome MDR

Many flavonoids are reported to either increase ROS or inhibit the antioxidant enzymes, exhibiting MDR reversing potential.

**Baicalein** significantly induces ROS production on tumor necrosis factor-related apoptosis-inducing ligand (TRAIL) resistant prostate cancer PC3 cells, leading to TRAIL re-sensitization. The ROS scavenger catalase prevents TRAIL sensitization, indicting it a ROS mediated mechanism (137).

Nrf2, a transcription factor, works with kelch-like ECH-associated protein 1 (Keap1) and the antioxidant response element (ARE) as a cytoprotective response to endogenous and exogenous stresses caused by ROS via the up-regulation of antioxidant proteins (138). A higher level of Nrf2 and its target proteins contributes in Dox resistance in BEL-7402/ADM cells (139). **Chrysin** suppresses Nrf2 at both protein and mRNA levels to BEL-7402/ADM cells, sensitizing the cells to Dox. Moreover, Chrysin also increases the intracellular concentration of Dox (139). Glutathione (GSH) is a peptide that significantly reduces the damage caused by toxic xenobiotics and ROS (140, 141). By depleting 50 to 70% of intracellular GSH within 24 h, Chrysin potentiates the cytotoxicity of curcumin (a natural occurring compound that kills cancer cells) to PC-3 cells and human leukemia cell line HL-60 cells (142). This effect is also found in non-small cell epithelial cancer cell lines A549, H157, H460, and H1975 (143). Co-treating with Chrysin (5–30 μM) significantly enhances the sensitivity of the cells to Dox as compared to Dox alone. Mechanistically, Chrysin may facilitate GSH efflux as demonstrated in Brechbuhl's study (143).

**Wogonin** robustly induces ROS accumulation in A549 cells and further sensitize A549 cells to TRAIL-induced apoptosis *in vitro* and *in vivo*, which can be reversed by ROS scavenger butylated hydroxyanisole (BHA) and N-acetyl-L-cysteine (NAC) (144). Moreover, Wogonin suppresses nuclear translocation of Nrf2 by NF-κB inactivation and induces more intracellular ROS as shown in K562/A02 cells and in HepG2 cells (145, 146), enhancing the effects of Dox in K562/A02 cells. Apigenin also lowers the GSH level, which then increases ROS levels, resulting in cell death of ABCC1 over-expressing H69AR-drug selected and HeLa/ABCC1-transfectant cells (147). Through similar mechanisms, Wogonin may enhance the efficacy of (1) cisplatin in cisplatin resistant HNC cells (149), (2) Dox in its resistant human myelogenous leukemia K562/A02 cells (99), and MCF-7/DOX cells (148).

**Luteolin**, 3′,4′,5,7-Tetrahydroxyflavone, which is abundant in leaves and aromatic flowering plants, possesses inhibitory effects to pancreatic cancer cells (149), colorectal adenocarcinoma LoVo cells and in drug-resistant LoVo/Dx cells (150), etc. Luteolin is also able to inhibit Nrf2 markedly and enhance the cytotoxicity of cisplatin in cholangiocarcinoma KKU-100cells (151). By inhibiting Nrf2 (152), luteolin may enhance the efficacy of (1) oxaliplatin in oxaliplatin-resistant colorectal cancer cell lines HCT116-OX and SW620-OX cells (153), (2) bleomycin, Dox in A549 cells (154), and Dox in MDA-MB 231 cells (155).

Other flavonoid that modulate ROS includes **Galangin**, purified from the *Alpinia galangal* root, exhibits collateral sensitivity (156), a phenomenon where one compound shows better inhibitory effects to resistant cancer cells over sensitive cells (157).

The iso-flavonoid **Genistein** and **7,3′,4′-trihydroxyisoflavone**, one of the major metabolites of daidzein found in fruits, nuts, and soy-based food (158), also exhibits bioactivity to Nrf2 and ROS. Genistein down-regulates the level of methylation in the Keap1 promoter region, which inhibits the transcription of Nrf2 to the nucleus, resulting in the suppression of Nrf2-dependent antioxidant enzymes and up-regulation of ROS in A549 cells (159). Through this mechanism, Genistein significantly increases cell apoptosis in A549 cells when combined with radiation (159). Through the induction of ROS and by down-regulating ABC transporters ABCB1, ABCC1 and ABCC2, **7,3′,4′-trihydroxyisoflavone (**25 μM**)**, significantly increases the intracellular accumulation of epirubicin and attenuates epirubicin resistant in HeLa cells (160).

### Flavonoids That Regulate Hypoxia to Overcome MDR

Many flavonoids are found to regulate hypoxia to reverse MDR.

**Baicalein** suppresses the HIF-1α expression in 5-FU resistant gastric cancer AGS cells ny inhibiting the hypoxia-induced Akt phosphorylation, which finally leads to re-sensitizing 5-FU (161).

**Wogonin** decreases the expression of HIF-1α in human colon cancer cell lines HCT116 by inhibiting the PI3K/Akt signaling pathway. Through this mechanism, Wogonin enhances the cytotoxicity of Dox, cisplatin, paclitaxel to HCT116 cells (162).

**Quercetin** is another flavonoid that regulates HIF-1α, which consequently re-sensitizes Dox to Dox resistant breast cancer MCF-7/dox cells (120), and 4T1 cells (163), cisplatin and etoposide to HCT116 cancer cells (164).

### Flavonoids That Regulate CSCs to Overcome MDR

Many flavonoids have been shown to suppress the growth of CSCs.

**Baicalein** may selectively re-sensitize CD133^+^ tumor initiating CSCs (isolated from human liver tumors which exhibit drug resistance properties) to certain chemotherapeutics (36). Baicalein inhibits the SAR1B GTPase which is necessary for autophagy, a way cancer cells apply to avoid cytotoxic effects induced by chemotherapeutics (36). Furthermore, Baicalein works synergistically with the mTORC1 inhibitor in a patient-derived xenograft model of hepatocellular carcinoma via elimination of CSCs (165).

On human CD44^+^ prostate CSCs (isolated from human PC3 cells) which confer MDR, **Apigenin** is able to significantly enhance cisplatin's efficacy by down-regulating the mRNA expression of anti-apoptotic Bcl-2, sharpin and surviving, and up-regulating pro-apoptotic caspase-8 and p53 (166). On another two CSCs cells, glioblastoma multiforme U87MG and U373MG cells, Apigenin significantly suppresses the cell growth, clonogenicity, and invasiveness, three key factors that represent the self-renewal property of CSCs. Mechanically, Apigenin blocks the phosphorylation of c-Met and its down-stream targets, such as the transducer and activator of transcription 3 (STAT3), Akt and protein kinase mitogen-activated protein kinase (MAPK) (167).

**Wogonin** exhibits anti-CSCs effects, as shown on CD133 human osteosarcoma CSCs (168). Wogonin induces apoptosis, inhibiting the mobility by down-regulating the expression of metallopeptidase-9, leading to a halt in its renewal ability (168).

Other flavonoids that regulate CSCs are **Luteonin** and **Quercetin**. Luteonin is able to eliminate the CD44+/CD49f+ CSCs isolated from TNBC via ribosomal S6 kinase inhibition (169). Quercetin may suppress the self-renewal property of pancreatic cancer stem-like cells which is gemcitabine resistant via targeting β-catenin, restoring the sensitivity of gemcitabine *in vitro* and *in vivo* (170). Quercetin also inhibits the breast CSCs (171), colorectal CSCs and restores the sensitivity of Dox (172).

### Flavonoids That Regulate the Cell Cycle to Overcome MDR

Some flavonoids have been shown to regulate the cell cycle to overcome MDR.

The cyclin E2 mRNA and protein expression was higher in tamoxifen resistant MCF-7 cells compared with sensitive cells. **Luteolin** specifically inhibits the Cyclin E2 protein expression in resistant cells and exhibits a synergistic effect with tamoxifen (173).

By down-regulating cyclin D1, **Quercetin** significantly enhances the efficacy of Dox in TNBC cells (174), and cisplatin in ovarian carcinoma SKOV3 cells and osteosarcoma U2OS cells (175).

**Scutellarin**, an active flavone extracted from Erigeron breviscapus Hand-Mazz, down-regulates Cdc2, cyclin B1, two cell cycle related proteins, and induces G2/M arrest and apoptosis to PC3 cells, and restores the sensitivity of cisplatin (176).

### Flavonoids That Regulate Autophagy to Overcome MDR

In Dox resistant BEL-7402/ADM cells, **Apigenin** significantly enhances the sensitivity of Dox, induces miR-520b expression and inhibits autophagy-related protein 7 (ATG7)-dependent autophagy *in vitro* and in hepatocellar carcinoma xenografts model (177).

Treatment of ovarian cancer cells with cisplatin may elevate poly [ADP-ribose] polymerase 1 (PARP-1), which is important for cell survival by regulating autophagy. **Leteolin** can inhibit PARP-1 at both the mRNA and protein level, and suppress autophagy, restoring the sensitivity to cisplatin (178).

Another flavonoid **Icaritin** can inhibit epirubicin-induced autophagy which may cause epirubicin resistance, and acts synergistically with epirubicin to suppress the proliferation of BT5637 and T24 cells (179).

### Flavonoids That Regulate EMT to Overcome MDR

As shown in pemetrexed-resistant NSCLC A549-R, H358-R, H460-R cells, EMT pathway promotes the MDR profile. **Kaempferol** is able to inhibit EMT signaling, rendering the resistant cancer cells susceptible to pemetrexed (180).

Another study indicates that EMT contributes in paclitaxel-resistance in ovarian cancer X10 and X22 cells. **Luteolin** at non-cytotoxic dose can reverse EMT, and re-sensitize the two cells to paclitaxel (181).

### Flavonoids That Regulate Critical Enzymes to Overcome MDR

#### STAT3

**Chrysin** selectively decreases the STAT3 phosphorylation to A549 cells, and re-sensitizes A549 cells to TRAIL (182).

On cisplatin-resistant lung cancer A549/DDP cells, combination of **Galangin** and cisplatin suppresses the cell proliferation through inhibiting p-STAT3 and anti-apoptotic Bcl-2 and increasing pro-apoptotic Bax and Bid. This combination also exhibits potency in mice xenograft models (183).

Similar results are also found by pretreatment of Quercetin which significantly enhances the cytotoxicity of cisplatin in an ovarian cancer cell line by suppressing STAT3 phosphorylation and Bcl-2. In a xenograft mouse model of ovarian cancer, Quercetin enhances the antitumor effect of cisplatin (184).

#### p53

Li et al. reported that a combination of **Chrysin** and cisplatin increases p53 phosphorylation and accumulation by activating ERK1/2 in HepG2 cells, leading to significant apoptosis, evidenced by the over-expression of pro-apoptotic proteins Bax, death receptor 5 and the inhibition of the anti-apoptotic protein Bcl-2 (185).

**Apigenin** is reported to elevate p53 and up-regulate certain pro-apoptotic proteins, which may increase cisplatin-induced DNA damage and apoptosis of A549 and H1299 cells (186).

**Quercetin** may potentiate 5-FU in 5-FU resistant HCT15 cells (which harbor a p53 mutation) by increasing p53 expression and activating the apoptotic mitochondrial pathway (187).

Through activating the extracellular signal-regulated kinases (ERK)-mediated p53 pathway, **Scutellarin** is capable of sensitizing A549/DDP cells to cisplatin *in vitro* and *in vivo* (188).

#### NF-κB

By decreasing the activity and of NF-κB, Fisetin increases the expression of death receptor TRAIL-R1, strengthening the apoptosis induction effects of TRAIL to TRAIL-resistant androgen-dependent LNCaP cells (50).

**Genistein** is able to suppress NF-κB, potentiate cisplatin, docetaxel, Dox or gemcitabine in various cancer cells including prostate, breast, lung, pancreatic and ovarian cancer cells (189–191). Similarly, genistein also enhances oxaliplatin in gemcitabine-resistant pancreatic cancer cells (192), gemcitabine in osteosarcoma cells, cisplatin in medulloblastoma cells (193), arabinoside in acute myeloid leukemia cells (194), and arsenic trioxide in human hepatocellular carcinoma cells *in vitro* and *in vivo* (195), suggesting it a promising reversal agent.

The activation of NF-κB contributes to TRAIL resistance of prostate cancer LNCaP and DU145 cells. **Biochanin A** significantly augments the cytotoxicity of TRAIL in these two cell lines, and it sensitizes the TRAIL-resistant LNCaP cells through NF-κB inhibition, leading to an up-regulated death receptor TRAIL-R2, and the disrupted mitochondrial membrane potential (196).

**Tectorigenin**, isolated from the flowers of *Pueraria thunbergiana* (197), exhibits re-sensitizing effects on paclitaxel-resistant ovarian cancer cells MPSC1(TR), A2780(TR) and SKOV3(TR) (198). Tectorigenin inhibits NF-κB nuclear translocation and its target genes, such as FLIP, XIAP, Bcl-2, Bcl-xL, and COX-2, all of which are known to be associated with MDR. Consequently, Tectorigenin enhances the inhibitory effect of paclitaxel in these three paclitaxel-resistant ovarian cancer cells (198).

### Flavonoids That Regulate Other Key Enzymes Regulated to Overcome MDR

It should be noted that apart from the aforementioned three key players in MDR, flavonoids also regulate other enzymes, e.g., **Fisetin** and **Genistein** regulate Akt to suppress Irinotecan and Oxaliplatin resistant CPT11-LoVo cells *in vitro* and *in vivo* (199), and enhance the cytotoxicity of cisplatin in A549 cells (200), respectively. **Fisetin** decreases the phosphorylated MAPK to increase the sensitivity of cisplatin-resistant A549-CR cells to cisplatin (201) and erlotinib-resistant lung cancer cells to erlotinib (202). **Apigenin** inhibits adenine nucleotide translocase-2 (ANT2) to enhance the efficacy of TRAIL to prostate cancer DU145 and LNCaP cells (203). **Luteolin** inhibits vaccinia-related kinase 1 (VRK1) to enhance the efficacy of cisplatin in esophageal squamous cell carcinoma (204). **Quercetin** suppresses the proliferation of tamoxifen resistant breast cancer TAMR-MCF-7 cells by inhibiting the expression of Pin1, vascular endothelial growth factor (VEGF), HIF as well as activator protein-1 (AP-1) (205). **Quercetin** also re-sensitizes enzalutamide to enzalutamide-resistant prostate cancer cells to *in vitro* and *in vivo* by inhibiting Androgen receptor splice variant 7 (AR-V7) (206). **Genistein** abolishes the increased cyclooxygenase-2 (COX-2) to 5-FU resistant HT-29 colon cancer cells (207) and induces the cleavage of Bid to TRAIL resistant human hepatoma cells (208), exerting its sensitizing effects. **Chrysin**, **Apigenin**, **Luteolin**, **Quercetin** and **Genistein**, also regulates the ubiquitin-proteasome pathway to overcome MDR in various chemotherapeutic drugs (209).

Furthermore, **Chrysin** may inhibit the pro-inflammatory mediators including interleukin-6 (IL-6) and the aldo-keto reductases superfamily (AKR1C1/1C2) expression, re-sensitizing cisplatin and Dox in NSCLC (210).

**Apigenin** also targets Axl and Tyro3 receptor tyrosine kinase (211), and impacts mitochondrial membrane potential (212), antagonizes Mcl-1 upregulation (213), or acts as an anti-estrogen and a protein kinase inhibitor (214) to sensitize certain chemotherapeutics.

**Kaempferol** may also enhance the efficacy of TRAIL in human ovarian cancer cells OVCAR-3 and SKOV-3 cells (215), and U251 and U87 glioma cells (216) via JNK/ERK-CHOP pathway and induction of proteasomal degradation of survivin, respectively.

**Wogonin** may also regulate AKR1C1/1C2 (210), and tumor necrosis factor-α (217). Wogonin increases Dox sensitivity through the down-regulation of the IGF-1R/AKT signaling pathway in human breast cancer (218), and increases the activity of sorafenib to human hepatocellular carcinoma cells by potentiating apoptosis and inhibiting autophagy (219).

**Quercetin** may potentiate the effect of fludarabine and ABT-737 against CLL via Mcl-1 inhibition (220), enhance the efficacy of (1) Dox in the Dox resistant prostate cancer (PC)3 cell line (PC3/R) by down-regulating c-met (221) and Dox resistant human leukemic MDR K562/ADR cells by regulating JNK/MAPK (222), (2) TRAIL to pancreatic cancer cells through JNK-mediated cFLIP turnover (223), (3) tamoxifen in tamoxifen-resistant breast cancer cell line (MCF-7Ca/TAM-R) by up-regulating ERα and down-regulating Her-2 (224), etc.

## Discussion and Future Perspective

An increasing body of studies have suggested that through single or combinational administration, flavonoids, and iso-flavonoids may work as sensitizing agents. While the major issue of mechanism study of flavonoids is the lack of specific targets and their acting mechanisms in surmounting, resistant cancer cells are still not understood properly (55). Currently, studies like those discussed above indicate that these flavonoids exert their anticancer efficacy through multiple mechanisms, and multiple targets, and it's quite clear that certain flavonoids may overcome MDR by regulating various aspects that contribute to MDR. Therefore, they could be characterized as multi-functional natural compounds rather than multi-targeting agents (54, 105, 225, 226).

Flavonoids tend to target lipid bilayers and modify the membrane physicochemical properties to exert their bioactivities (227–230). As demonstrated in Ingólfsson et al.'s study, phytochemicals of different structures (polyphenols including flavonoid) could alter lipid bilayer properties as they localized on the bilayer/solution interface. Through a similar action, they also regulated bio-functions of diverse membrane proteins, suggesting that their actions may be due to the common, membrane bilayer-mediated mechanism (231). Therefore, we conclude that flavonoids do not function as specific regulators of target proteins, but rather as multi-functional agents that negatively regulate the key factors contributing to MDR as well as to other diseases.

To reverse MDR, these flavonoids may regulate many targets. First, the flavonoids may regulate ABC transporters, such as ABCB1, ABCG2, ABCC1, etc. They not only inhibit the efflux effects of these transporters to many conventional chemotherapeutics, but also inhibit the expressions. Docking studies indicate that they may bind to NBDs of ABCB1 (83). To date, no ABC transporter regulators have been approved by the FDA because of severe adverse effects. Natural products hold promise to be of lower toxic agents, given that many of the flavonoid regulators already serve as dietary supplements.

Second, as polyphenolic compounds, many flavonoids may work as ROS modulators (18) as they affect the status of ROS level in cancer cells (232). Under different dose, they may work either as ROS scavengers or inducer. To overcome MDR, they preferably work as inducers which induce more ROS production that can reach to the toxic threshold to activate apoptosis (18). Major players in maintaining balanced ROS in cells include Mrf2, GSH, both of which can be inhibited by certain flavonoids to exert their re-sensitizing effects.

**Figure 3:**
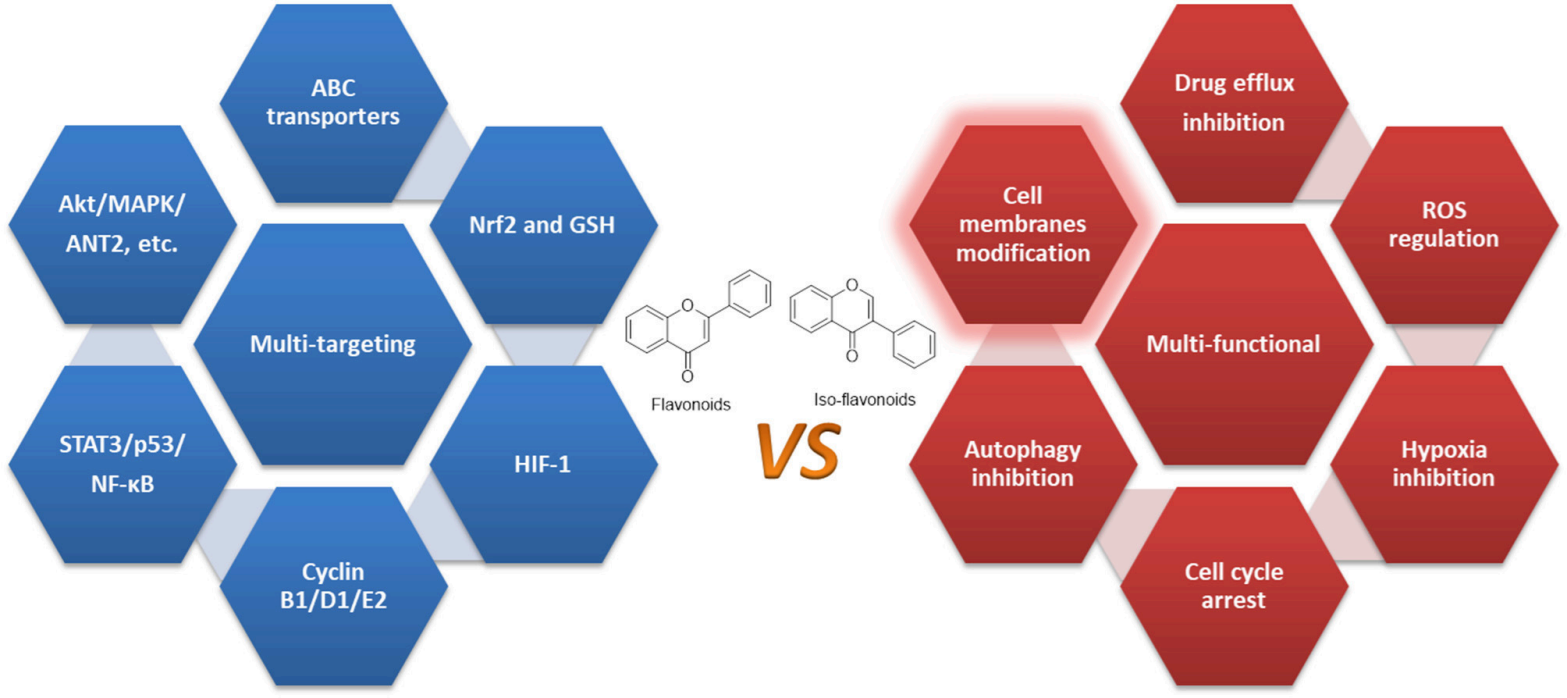
Multi-functional flavonoids in overcoming MDR.

Third, they may also regulate HIFs, cell cycle, CSCs, autophagy, and critical enzymes such as STAT3, p53, and NF-κB, confirming their multi-functional property as summarized in Figure 3 and Table 1.

**Table 1:** Summary of the flavonoids with MDR reversal effects and their application.

**Compound name**	**Functions**	**Application**	**References**
Chrysin	ABC transporters regulation ROS induction STAT3 inhibition p53 inhibition	Sensitizing mitoxantrone Sensitizing Dox, curcumin Sensitizing TRAIL Sensitizing cisplatin	(70) (139, 142) (182) (185)
Baicalein	ABC transporters regulation ROS induction Hypoxia suppression CSCs inhibition	Sensitizing docetaxel, paclitaxel Sensitizing TRAIL Sensitizing 5-FU Sensitizing mTORC1 inhibitor	(77, 78) (137) (161) (165)
Apigenin	ABC transporters regulation CSCs inhibition Autophagy inhibition p53 inhibition	Sensitizing docetaxel, doxorubicin Killing resistant CSCs Sensitizing Dox Sensitizing cisplatin	(84–86) (166, 167) (177) (186)
Acacetin	ABC transporters regulation	Sensitizing Dox, SN-38	(91, 92)
Wogonin	ABC transporters regulation ROS induction Hypoxia suppression CSCs inhibition	Sensitizing Dox Sensitizing Dox, cisplatin Sensitizing Dox, cisplatin, paclitaxel Killing resistant CSCs	(99) (147, 233) (162) (168)
Kaempferol	ABC transporters regulation EMT suppression	Sensitizing cisplatin Sensitizing pemetrexed	(107) (180)
Galangin	ROS induction STAT3 inhibition	Collateral sensitivity Sensitizing cisplatin	(157) (183)
Naringenin	ABC transporters regulation	Sensitizing daunomycin	(112)
Luteolin	ROS induction CSCs inhibition Cell cycle regulation Autophagy inhibition EMT suppression	Sensitizing oxaliplatin, Dox Killing resistant CSCs Sensitizing tamoxifen Sensitizing cisplatin Sensitizing paclitaxel	(151, 153–155) (169) (173) (178) (181)
Quercetin	ABC transporters regulation Hypoxia suppression CSCs inhibition Cell cycle regulation p53 inhibition	Sensitizing Dox, paclitaxel Sensitizing Dox, cisplatin, etoposide Sensitizing gemcitabine, Dox Sensitizing Dox, ciaplatin Sensitizing 5-FU	(119–122) (120, 163, 164) (170, 172) (175) (187)
Rutin	ABC transporters regulation	Sensitizing paclitaxel	(117)
Fisetin	ABC transporters regulation NF-κB inhibition	Sensitizing cabazitaxel, paclitaxel Sensitizing TRAIL	(123, 124) (50)
Scutellarin	Cell cycle regulation p53 inhibition	Sensitizing cisplatin Sensitizing cisplatin	(176) (188)
Icaritin	ABC transporters regulation Autophagy inhibition	Sensitizing Dox Sensitizing epirubicin	(128) (179)
Genistein	ABC transporters regulation ROS induction NF-κB inhibition	To be explored Sensitizing radiation Sensitizing cisplatin, Dox, gemcitabine	(131–133) (159) (189–191)
Biochanin A	ABC transporters regulation NF-κB inhibition	Sensitizing mitoxantrone Sensitizing TRAIL	(136) (196)
7,3′,4′-Trihydroxyisoflavone	ROS induction	Sensitizing epirubicin	(160)
Tectorigenin	NF-κB inhibition	Sensitizing paclitaxel	(198)

Furthermore, certain flavonoids exhibit collateral sensitivity, a phenomenon where one compound shows selectivity to kill resistant cancer cells over sensitive cells (157), such as Galangin and Chrysin (156), apigenin dimer (234), and another flavonoid desmosdumotin B (235), making the flavonoids more appealing agents in treating resistant cancers.

In addition, some of these flavonoids are now under clinical trials to treat certain cancers, such as Apigenin (NCT03139227), Quercetin (NCT03476330, NCT02989129, NCT01912820, NCT01538316), Icaritin (NCT01278810, NCT01972672, NCT02496949). Further positive results will surely entice more researchers to develop them as drug candidates, e.g., MDR reversal agents.

One issue in this research is that most studies are conducted *in vitro*, so further *in vivo* studies are warranted. Given that many of the flavonoids are used as ingredients in dietary supplements, their anticancer/sensitizing efficacy could be more readily determined in humans (236–238). The natural products in flavonoids represent novel treatment strategies to overcome MDR in cancer, and structural modifications of these compounds should be of interest for medicinal chemists. Indeed, many flavonoids derivatives have been developed to suppress resistant cancer cells, such as Chrysin acyl derivatives against drug-resistant human cancer cells (MES-SA/DX5, LoVo/DX) (239), nitro Genistein derivatives modified by nitro groups against cisplatin-resistant human ovarian cancer A2780 cells (240), Quercetin-glutamic acid conjugate (241), and apigenin-based flavonoid dimers (242) against P-gp overexpressing cancer cells, selenium-containing Chrysin and Quercetin derivatives against cisplatin resistant cancer cells (243), Quercetin-3-methyl ether against lapatinib-resistant breast cancer cells (244), etc. These studies provide crucial information for new drug discoveries based on flavonoids.

## Conclusion

Dietary natural flavonoids possess multiple bioactivities including anti-cancer and chemo-sensitizing effects. Studies show that they inhibit certain ABC transporters, antioxidant enzyme Nrf2 and its related enzymes and regulate HIFs, CSCs, autophagy, EMT, etc., to exert their sensitizing effects, suggesting that they are multi-functional molecules.

## Author Contributions

QY, FH, and R-WJ conceived the topic. QY, KL, QS, QL, JH, FH, and R-WJ wrote the paper.

### Conflict of Interest Statement

The authors declare that the research was conducted in the absence of any commercial or financial relationships that could be construed as a potential conflict of interest.
